# Donkey Ownership Provides a Range of Income Benefits to the Livelihoods of Rural Households in Northern Ghana

**DOI:** 10.3390/ani11113154

**Published:** 2021-11-04

**Authors:** Heather C. Maggs, Andrew Ainslie, Richard M. Bennett

**Affiliations:** School of Agriculture, Policy and Development, University of Reading, Reading RG6 6AR, UK; a.m.ainslie@reading.ac.uk (A.A.); r.m.bennett@reading.ac.uk (R.M.B.)

**Keywords:** income generation, gender, children, donkey hire, human-animal interaction

## Abstract

**Simple Summary:**

Until recently, the important contributions donkeys make to the daily lives of millions of people around the world have been overlooked. Global donkey populations are under threat from increasing demand for their skins, a key ingredient in a traditional Chinese medicine called e’jiao. The aim of this research was to study the role of donkeys in rural households in northern Ghana. We wanted to know how donkeys support families in making a living, especially their use by women and children. We found that donkeys are highly valued by their owners. Donkeys help reduce the hard physical work of many farming and domestic activities and can also be rented out to generate income. There are actually more methods available to earn money from the family donkey than previously known. Females from donkey-owning households advised us that their donkey can provide between 30–60% of their total income. Children can also play a key role accompanying their donkey when it is hired out for cash. Donkeys are certainly important to their owners, who describe them as priceless. This research adds to our understanding of the impact of the e’jiao industry, by detailing the considerable value of live donkeys to poor farming households.

**Abstract:**

Donkeys provide important resources and benefits for millions of people worldwide. However, global donkey populations are under increasing pressure from the growing demand for a traditional Chinese medicine, e’jiao, made from donkey-skin. The objective of this reflexive, qualitative thematic analysis was to examine the role of donkeys with 262 participants in northern Ghana and how donkeys contribute to livelihood outcomes, especially their use by women and children. Data were collected from four surveys, 12 in-depth interviews and 84 daily time budgets with the same participants, plus 16 focus groups, during one wet and one dry season across 2018-19. Uniquely, boys and girls between the ages of 10–16-years old were interviewed. Donkeys are highly valued by their owners as they play a valuable role in providing a pathway out of ultra-poverty. Donkeys’ contributions to livelihoods are significant and more complex than previously understood and documented in the literature. Donkey ownership confers up to six different income benefits in comparison to non-donkey owners. Female owners of donkeys reported that donkeys can contribute between 30–60% of their income. Children of both sexes can play an important role in the efficient deployment of one of these income generating activities.

## 1. Introduction

“Anytime I don’t have money, I use the donkey to work and earn money at the end of the day. I can make at least 50.00 GH¢ (£6.48) and I can use that money to buy a female goat and it will reproduce”

Kahiau, (RID27M67).

**Figure 1:**
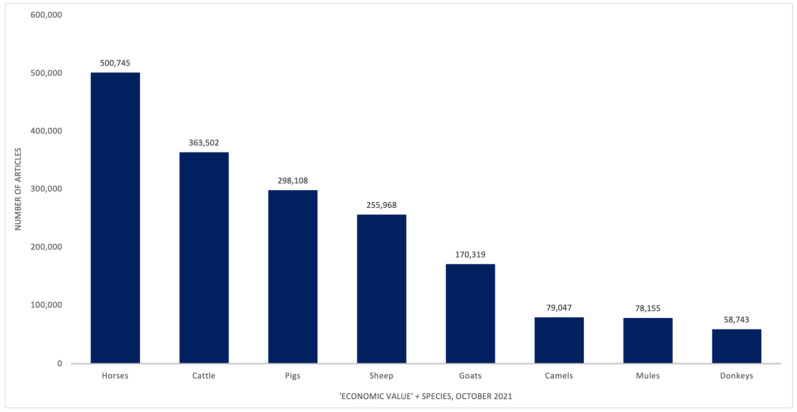
Number of key publication types by the search term “economic value AND species” in the ProQuest database, 11 May 2021. Note: total figure includes scholarly journals, dissertations/theses, books, newspapers, wire feeds, magazines, trade journals and reports.

The aim of this research was to understand the extent to which donkeys underpin the livelihoods of donkey owners in rural north Ghana, especially their use by women. Native to northeast Africa [1], there are a range of literatures relating to different aspects of *Equus africanus asinus*. As well as the clinical veterinary, health and welfare literatures [2,3,4,5,6] these include draught animal power (DAP), traction and transport [6,7,8,9], donkeys and livelihoods [8,9,10,11,12], donkey commodities [13,14,15,16] and donkeys and gender [8,17,18,19,20,21]. There is also a growing literature on the increasing demand for donkey hides, a key constituent of a traditional Chinese medicine (TCM) called e’jiao [3,16,18,22,23,24,25,26]. However, in comparison to other economically important species in Majority World agricultural production, donkeys have been under-researched and under-valued. One of the clearest demonstration of donkeys being under-researched across donkey literatures is McLean and Gonzalez [26] p. 26, where the authors investigated the number of papers focused on donkeys from 1880 to the present. Donkeys entered the record in 1896; one paper on average was published per annum until ~1980, when the numbers of papers began to increase with a steady increase from 2000, reaching a “new record (… in 2017 of) fifty seven articles dealing with different topics involving donkeys or their products” (*ibid*). A search to compare the number of relevant articles on economic value and particular species was undertaken in the ProQuest database (Figure 1, 15 October 2021). Using the search term “economic value AND species” demonstrates donkeys are under-researched in comparison with various livestock species in Majority World countries. FAO data [27] indicate that in 2018 the global population of mules was lower than that of donkeys, at ~9.3 m compared with ~53 m respectively. Donkeys are less researched than their hybrid relation, at 3 papers per 10,000 donkeys, versus 20 papers per 10,000 mules, supporting McLean and Gonzalez’s 2018 findings.

**Figure A1:**
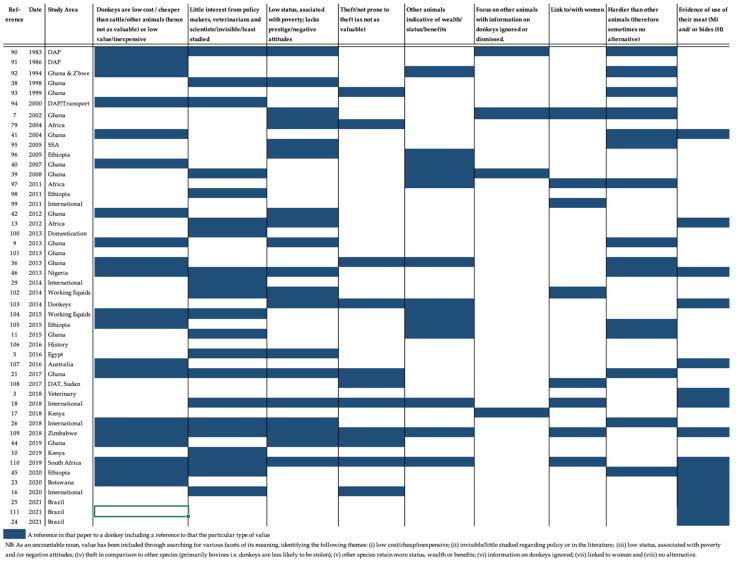
References to donkeys being under-valued in the literature [90,91,92,93,94,95,96,97,98,99,100,101,102,103,104,105,106,107,108,109,110,111].

One of the first animals to be domesticated [28], donkeys have provided essential services and potential routes out of poverty [18,20] to millions of people for millennia. However, prior to 2017, the dominant narratives in the literature were that donkeys were associated with people thought of as poor [5,18,21,29], under-valued in comparison to other livestock species, and largely invisible to development professionals, policy officials and governments (Figure A1, Appendix A). The embedded perception of donkeys in the literature as under-valued began to change with the publication of a report by the UK equine charity, the Donkey Sanctuary (TDS) in 2017 [30]. Entitled “Under the Skin,” the report was based on the growing awareness of the growth in the donkey hides trade (DHT) to supply the demand for e’jiao. E’jiao manufacturers reported their need for four to ten million donkey skins a year to meet the demand for e’jiao [31], leading to concerns regarding the potential impacts on global donkey populations and their welfare. Information about the DHT from the grey literature framed initial debate [32,33] including the BBC [34]. Since 2018 there have been increasing numbers of rigorous, systematic academic papers on donkeys, their welfare, the DHT and the services they offer to the communities who rely on them [3,10,16,18,22]. Within the donkey-specific literature this also includes McLean and Gonzalez [26] p. 25, who acknowledge that until recently donkeys had a low monetary value and consider the demand for e’jiao is contributing to the “increased value of donkeys [seen] in many developing regions of the world”.

Post-2017, the value of donkeys is increasing, ironically underpinned by the increasing prices of donkeys driven by the demand for e’jiao. Within this context, this research is in the vanguard of the increasing number of academic papers on donkeys, their welfare, the DHT and the services they offer to the communities who rely on them. The objectives this paper set out to address were to examine the role of donkeys in rural households in northern Ghana and their contribution to livelihood outcomes, especially their use by women and children. Donkey populations on the continent today are concentrated in Sahelian West Africa, Ethiopia and Egypt [35]. Ghana’s ~14,910 donkeys [27] are mostly found in the state’s northern regions [36]: Upper West Region (UWR), Upper East Region (UER) and Northern Region. The soils and agriculture of northern Ghana suit the use of both donkeys and bullocks for ploughing because the soils: “are relatively loose, dry and sandy. In the South of Ghana, the wet soil is too heavy and there are cattle diseases, making AT [animal traction] unviable. Traction animals are mainly used for “ploughing and transport,” [37] p. 2. Within this broad categorisation, donkeys are primarily used for domestic chores and farming activities throughout the agricultural cycle, including ploughing, weeding and land preparation, as well as the transport of water, firewood, building materials and farm and market produce [36]. 

**Figure 2:**
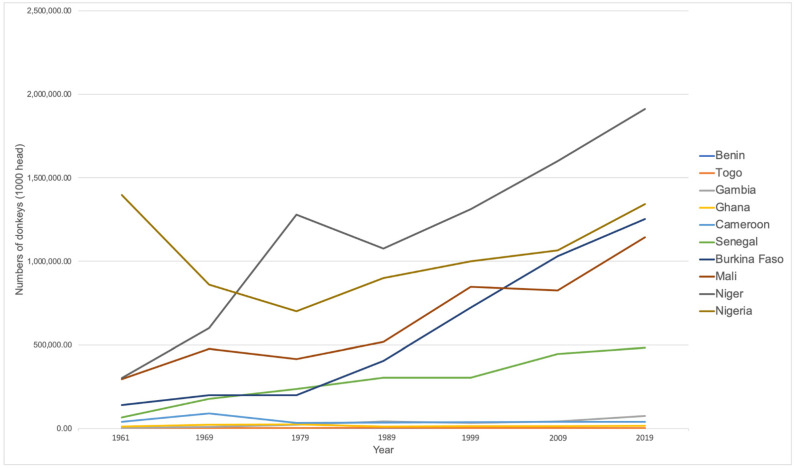
West African donkey populations 1961–2019 (FAO). Note: the quality of the data differs and includes official estimates, FAO estimates, unofficial figures, FAO data base and FAO imputation methodology. FAO. Source: FAOSTAT/asses, accessed 7 March 2021.

Despite a comparatively low number of donkeys in comparison to neighbouring West African states (Figure 2), research in relation to donkeys has been undertaken in Ghana. This includes studies of donkey behaviour [38]; their role in DAP [39,40,41]; livelihoods [40]; transport [42]; increased productivity [40,43]; food security [11]; and production and management [44]. Braimah et al., [9] p. 22 reported that the value to donkey owners (DOs) of ploughing and transportation assistance in Ghana’s UER appears to be the time and drudgery donkeys save both genders, particularly women, in undertaking domestic and agricultural chores. Several studies [10,45,46] interviewed non-donkey owners (NDOs). The term donkey-cart transport is a more accurate description, as donkeys are not used as pack animals in either study site. Instead, they are harnessed to a cart or plough which has, according to Dietz et al., [43] p. 26 resulted in “a veritable explosion in the number of […] donkey carts in northern Ghana”. In a DAP study from 10 districts in northern Ghana, Houssou et al. [36] p. 1, reported that “Farmers primarily use the animals on their own farms and provide service to their neighbours on hiring basis for cash or in kind”. Other studies have also reported evidence of donkeys being hired to generate income [20,21,36,44,45]. Tuaruka and Agbolosu’s 2019 research, undertaken in Bunkpurugu/Yunyoo District in the Northern Region of Ghana, (199–389 km [47] to the East of the present study’s field sites), concluded that the transportation of water was “the animals’ key role, carting of goods to market and farm implements to and from farms was the second most important role. The least role was for income generation” [21] p. 1. While evidence of the DHT has been reported in Ghana [30,48,49,50] it was not mentioned in Tuaruka and Agbolosu’s paper (2019) [44] p. 4. The authors report that an implication from their findings was the likely extinction of donkeys as work animals in the district, because of the trials facing the communities.

The research questions underpinning this study set out to examine what relationship donkeys play in the provision of livelihoods in northern Ghana and whether women—at least in part—are relieved of onerous tasks by donkeys, thus freeing their time to undertake other activities. In the next section, we review our materials and methods. In Section 3 the results of the analysis are presented, using short case studies to illustrate six different income benefits derived from the ownership and hire of donkeys. The role played by children in these benefits was also evaluated. Essential Activities of Daily Living (EADLs), adapted and developed from the gerontological concept, “the Activities of Daily Living” in the Minority World, [51], were used to aid understanding and consider how donkeys help participants. The results are discussed in Section 4 and Section 5 considers their implications. 

This paper argues that the contribution donkeys make to the lives of rural households in northern Ghana has been underestimated. DOs and NDOs were both interviewed, with NDOs offering additional insights into gender differences in how donkeys contribute to livelihoods. Consistent with the literature, DOs do use their donkeys to generate income through direct hire opportunities. However, this contribution is not only more complicated and nuanced than previously thought, but children can play a key role in the efficient deployment of one of these opportunities. This study also reports previously unrecognised, indirect methods DOs can use their donkeys to make money, requiring complex time management for effective household resource allocation. This often involves children. Donkey ownership can also provide DOs health and wellbeing benefits and underpin increasing petty trading opportunities for women. Notwithstanding a degree of social desirability bias, this paper therefore advances the understanding of donkeys as an important provider of DAP in northern Ghana. This study attempts to understand and document the value of donkeys to the livelihoods of two communities in northern Ghana. A gendered perspective highlights that the ownership of donkeys plays a valuable role in providing a pathway out of ultra-poverty. 

## 2. Materials and Methods

### 2.1. Preliminary Considerations

The study’s approach ensured that respondents were not viewed as research subjects, but as participants: their feelings, attitudes, thoughts and perceptions were sought to gain “knowledge […] arrived at through sense-making and meaning” [52] p. 50. A recognition of insider/outsider concerns, power dynamics and language underpinned data collection, leading to a reflexive qualitative thematic analysis [53]. All donkeys were owned by men. No child or female respondent personally owned a donkey at either study site. Throughout this paper the terms DO and NDO are therefore used as a shorthand for children from donkey- or non-donkey owning families. 

### 2.2. Data Collection and Study Location

Research was conducted in the Fielmon (UWR) and Gia (UER) communities in northern Ghana [54] during a dry and wet season: December 2018 and July 2019 respectively. Choice of study locations was finalised with the assistance of colleagues at the University for Development Studies (UDS), Ghana. UDS also provided two teams of experienced, multilingual research assistants for each study site, who were trained in the project’s aims and objectives by the lead researcher. 

**Figure 3:**
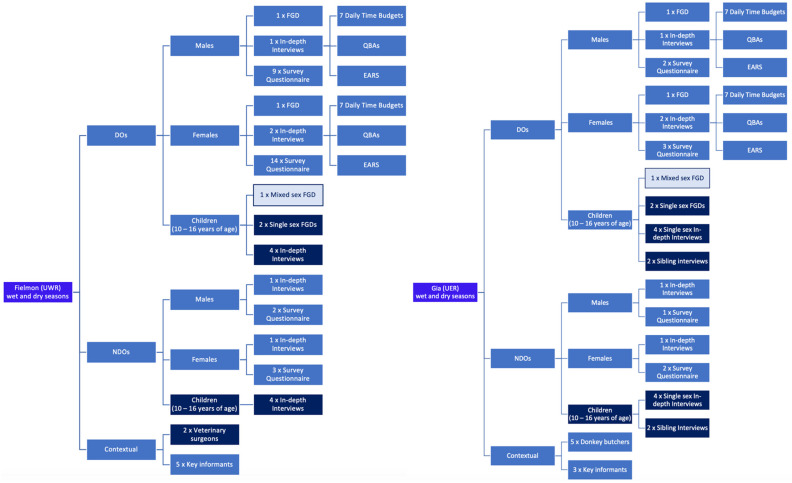
Study Research Instrument Schedule: the number of each research instrument conducted in each study site, with which audience.

Focus group discussions (FGD) and in-depth interviews (INDI) were undertaken with adult and children DOs and NDOs. In addition, a survey questionnaire was undertaken with male and female DOs and NDOs. DO INDIs also undertook seven daily time budgets (7DTB), charting their donkeys’ activities each evening for seven consecutive days This data was disaggregated by the age and gender of senior household members, including the eldest children of both sexes. These budgets were completed on seven consecutive evenings in both December 2018 (first visit) and June 2019 (second visit). Unstructured contextual interviews with key informants (*n* = 14) were held in both study sites (Figure 3).

The numbers of individuals involved in each research activity are outlined in Figure 3 and Table 1, with 262 participants in total. Copies of all research instruments are included in the supplementary material provided. All adult FGD respondents were from donkey-owning households: INDIs and questionnaires were completed across both study sites with the same adult DOs and NDOs across field visits, although no child was interviewed more than once. In addition, the Donkey Sanctuary’s Equid Assessment Research and Scoping (EARS) tool [55] and Qualitative Behavioural Analysis (QBA) welfare assessments [56] were undertaken on all donkeys from DO questionnaire and INDI respondents, also repeated across both seasons. All research was conducted in the local language: Dagaare (Fielmon) or Kasem (Gia). The research assistants translated and transcribed all recordings and provided transcripts in English. Male and female adults and children from NDO households were interviewed to provide context and provide additional, comparative insights. Innovative aspects of this study included (i) the research undertaken with children and their donkeys; (ii) interviewing the same adult DO and NDOs and assessing their donkeys across the two seasons and (iii) the 7DTBs completed by all DO INDIs of their household’s donkey-related activities for a seven-day period, repeated during each field visit.

### 2.3. Ethical Considerations

Key ethical issues considered prior to confirming the decision to research the voice of children included: protection from harm [57]; and payment dilemmas, power disparities and privacy guarantees [58,59]. All names used in this paper are pseudonyms for this reason, although where quoted, each respondent is identified by their unique respondent identification number (RIDNo), including an F or M to identify their gender and their age if known, with a figure. Potential translation issues [60] were taken into account to ensure that all adults and children had full understanding of their agreement to participate and how consent and assent could be captured. Ultimately, it was felt that as the children’s inalienable rights could be safeguarded, they had a right to express their opinions [61,62]. 

### 2.4. Ethics Approval and Sampling

The adult element of the study was approved by the University of Reading’s School of Agriculture, Policy and Development ethics committee: Ref:00809P, November 2018 and Ref: 001026, April 2019). The University of Reading’s Research Ethics Committee: Ref: UREC 18/52, December 2018 and Ref: UREC 19/44 April 2019 approved the research involving children. Purposive sampling was undertaken to recruit both DOs (*n* = 29) and NDOs (*n* = 12) adults across both study sites. The same research and consent/assent protocols were followed during both field visits. All the FGDs with children were oversubscribed: children drew lots as to who participated. Copies of all research instruments are included in the supplementary material provided.

### 2.5. Adult Research Instruments

Data in field were captured on paper using DO and NDO versions of survey questionnaires, INDI questions and discussion guides. To help overcome the issue of memory recall and aid data triangulation, the concept of the 7DTB was developed. The EARS and QBA assessments data were captured on prescribed electronic proformas. Unstructured contextual interviews (*n* = 14) were held in both study sites (Figure 3).

### 2.6. Children’s Research Instruments

Appropriate child-friendly data collection methods [63] were designed to elicit information on donkeys, based on the aims of the study. A child-friendly discussion guide was developed, designed to be fun and enjoyable for the children, which included: a post-it/graffiti wall; story board; draw and tell; emotion cards; an animal calendar; picture recognition cards and mapping, although the post-it/graffiti wall was developed into a shout out and tell whilst in the field, to save time.

### 2.7. Data Analysis

All the datasets resulting from this research were imported into NVivo 12.6.0 for Mac. Combined, this data provided a substantial amount of rich and detailed qualitative data. However, NVivo software was not used to analyse the data. NVivo was used entirely as a data management tool because of concerns expressed by other researchers, who argue that a computer can quickly manage, organize and break down a set of qualitative data, but cannot analyse and interpret qualitative data as effectively as a human being [64,65,66,67]. Mather et al. argued in 2018 [68] p. 1 that “Digital analysis software packages such as NVivo do not fully scaffold the analysis process”. Once inputted, the data was analysed using the key steps of the six-phase, recursive reflexive thematic analysis as described by Braun and Clarke in 2006 [53] p. 87 outlined by the following six steps:

Step 1: Become thoroughly familiar with the qualitative data.

Step 2: Generate an initial code for each relevant unit of information.

Step 3: Identify the primary themes based on the initial coding of the units of information.

Step 4: Identify the subthemes, by coding the manifestations within each primary theme.

Step 5: Review and refine the coding of the primary themes and subthemes.

Step 6: Tabulate and interpret the results of the thematic analysis.

The researcher’s own intuition, perception and inductive reasoning was used to ensure the drawing of meaningful conclusions from this qualitative data, by implementing this six-step framework. Reliable themes were identified from patterns in the data, when related units of information were aggregated and appeared multiple times within each set of data [69]. All units of meaning were analysed at least twice, by the lead researcher, employing two different elemental coding methods across all research instruments. These were chosen to develop codes from the data in the language of participants, not the researchers, specifically “to honor children’s voices and to ground the analysis in their perspectives” [70] p. 71. Five themes were developed from the resultant thick analysis [71], using Saldaña’s (2015) streamlined codes-to-theory model for qualitative inquiry [70] p. 14. These were livelihoods, gender, children, community and donkey welfare. They were developed into concepts through distinguishing the connotations, nuances and shades of meaning expressed by the participants’ self-reported experiences, thoughts, beliefs, perceptions, attitudes and needs.

## 3. Results

**Figure 4:**
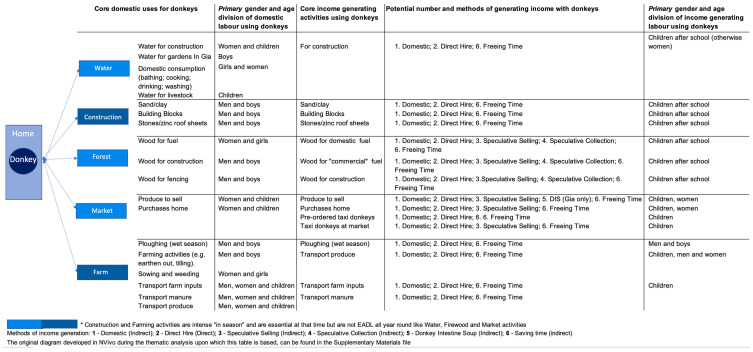
Division of age and gender related Essential Activities of Daily Living across domestic and “commercial” uses of donkeys.

Supporting the literature, male and female DO respondents from Fielmon reported that the principal role played by their donkey was to help them plough their land; in Gia, it was for transportation purposes [36,72]. Their secondary role in both communities was for transportation of various items, including occasionally being used to move musical instruments and chairs for community events. These roles primarily relate to the ability to transport greater loads in carts than a single person (Figure 4), and can be summarised into five core EADLs. These were the transportation of (i) water; (ii) construction materials; (iii) wood for fuel; (iv) produce to and from farm to home and home to market and (v) for farming activities including ploughing [36,72]. Farming activities and construction are not core on a daily basis, unlike water and firewood or weekly like key marketing activities, but are essential and intense at certain times of the agricultural year and when household and community building projects are in train. Although 48% of DOs (14/29 respondents) do hire out their donkey to make money, only a small minority of DOs mentioned income generation as the reason they owned a donkey, and this was never given as a core reason; it was always a secondary one.

As can be seen from the third column of Figure 4, there is a gendered difference to the EADLs undertaken by men, women and children. Fuel and water collection and marketing (petty trading) are dominated by women and children, especially girls when there is a domestic element to requirements. Ploughing, the collection of wood for construction and many farming activities are undertaken by men and boys, except for sowing and weeding, which are traditionally female-orientated activities [73] p. 90. Transportation of various items including farm inputs and manure to and from house to farm, and crops/produce from farm to home can involve the whole family. It is interesting that those EADLs associated mostly with male labour are those connected with seasonal/sporadic project work and those on a daily/weekly basis all year round with female-centred activities. This may help explain why the literature reports that donkeys reduce the drudgery of women more than men, although men do use their donkey to make money all year round.

### 3.1. A Market for Hiring out Donkeys and Carts

Those who purchase the services of a donkey and cart are primarily NDOs, although some households with only one donkey may hire or borrow a second from a friend or neighbour if the task requires two animals yoked together. Figure 4 (column four) shows NDOs mostly require help with the same core EADLs that DOs give for keeping donkeys: broadly, the transport of water, construction materials, wood, farm and market products, and ploughing. This has resulted in a fully-functioning, donkey-hiring market within both study communities: demand for the services of donkeys from NDOs, when they can afford them, fulfilled by DOs with ploughs and carts on the supply side. However, prices charged for the hire of donkeys varied, with some DOs saying they provide the services of their donkeys free of charge or at reduced rates to friends, family and neighbours [36,45]. As noted, money is not earned from a lone donkey, without additional equipment [22]. For effective income generation from donkeys, a cart or plough is required as well. Most DO households have these tools, as they are needed to use the donkey for domestic and agricultural EADLs, although one respondent reported that they owned a cart but were saving up for a donkey. Further benefits include the use of donkeys and carts reducing the amount of headloading DOs have to do, especially women. Potential health benefits include minimising axial stress [73,74,75,76,77,78]: the concentration of force or weight down through the long axis of the body.

### 3.2. Six Different Income Benefits from Donkey Ownership

Fifty-two per cent of DO respondents (15/29) across both Fielmon and Gia use their donkeys solely for domestic purposes. The 48% who do hire out their donkey to generate income revealed there are nuanced and numerous methods of generating money from doing so (Table 2). These can be classified into six different methods: one is a straightforward direct hire (method 2; 3.2.2.) with five indirect methods. DOs explained that they can make money just through owning a donkey, even if they do not specifically hire it out. This results in two cashless indirect categories. The first, method one, domestic (3.2.1) is where indirect financial benefits still accrue to DO families from the savings they make through not having to spend money hiring help in transporting goods or ploughing. The second opportunity, the conversion of time into money (Method 6, Freeing Time, 3.2.6) results from the time donkeys save women particularly, to undertake other tasks. The direct hire is the method outlined in the literature and saving time and saving transport costs from owning a donkey have also been noted [79]. The other three methods, previously unidentified in the literature, are all indirect, in which the role of the donkey has not been visible. These are: speculative sales; speculative collection and selling donkey intestine soup (DIS). That donkeys save their owners time is known, but respondents also reported the ability to take on paid work as a result of the time owning a donkey saves them. Five of these methods are available to all DOs, whilst selling DIS is only available in Gia. This method is location/culture specific as donkey meat is not eaten in Fielmon, where it is tabooed. However, it is open to both DO and NDO women. Credit has been excluded from this analysis, as only one female FGD respondent stated that using a donkey as collateral for credit was an available option in her community of Fielmon (RIDFW002), otherwise this was not mentioned.

Each method of income generation is described in more detail below, using individual case studies to illuminate how income generation strategies are embedded in the lives and livelihoods of respondents. Where relevant, the collection of wood from the bush is primarily used to describe each income generation method because both communities regard wood as a free resource, and the differences in the mechanics of each method outlined are then easier to compare. Quantities of wood are needed in every household on a daily basis for domestic use alone (heating water for bathing and cooking), as well as for construction and petty trading activities, like pito brewing (the local beer, the brewing of which is an exclusively female activity). Donkeys are the only animal in these communities that are used to transport goods, including the collection of wood. Cattle are also used to plough but respondents often preferred donkeys to motor tricycles for wood collection because they are cheaper and nimbler in accessing difficult to reach areas of forest. 

#### 3.2.1. Domestic Use of Donkeys for Non-Monetary Income Benefits (Cashless Indirect)

Income benefit one, is entitled “Domestic”. Owning a donkey and using it for the reduction of domestic and farming drudgery enables the family to save money on ploughing and transport costs, as this case study from Fielmon demonstrates: 

Case Study 1: the benefits of the domestic use of donkeys 

Jalia (RID25F) a 32-year-old from Fielmon has six children under 18: two boys and four girls. One boy and three girls are in school: the others are too young. They farm seven acres and own a mobile phone, a radio, a television and a motorbike, which her husband uses to commute to his tailoring shop in the village centre. He employs at least two boys. They used to own four donkeys but two died: they did not eat the meat but threw the carcasses away. In the past women were only responsible for sowing and weeding but now, when her husband is away Jalia undertakes nearly all other farming activities. She explained that “The donkeys are helping us a lot. If you have a donkey, you can do so many things. They helped us construct these houses. It is not easy begging people to help in these activities. The donkey helps us to cart sand, clay, roofing sheets, water and other building materials for the house construction … If you are to carry firewood five times a day the donkey would go once and carry more than that. Carrying the firewood five times can use about six hours, but the donkey would use about two hours … The donkey can plough an acre a day. This one acre would have [cost us] GH¢100 [80], (GB£15.31)”. However, Jalia explained that “some people are making a lot of income from the donkeys because they have children who can send them to work for others but we do not have somebody to work with the donkeys to earn us extra income. It only helps us to farm and cart farm produce home”. 

#### 3.2.2. Direct Income Generation Utilising Donkeys (Direct)

Income benefit two is called direct hire, where DOs make money from their donkeys through hiring them out to those in their community who require them to undertake a specific task, including ploughing. A date, time and activity are agreed between the two parties: it can be on the same day, or scheduled in advance: DOs can arrange the activity to suit, often out of school hours, for reasons cited below. In terms of the firewood example, DOs implied that their donkey and cart would be hired to go into the bush and collect firewood while they waited, although one NDO collected the firewood first and then hired a cart to help bring it home. The problem with this latter course is the danger of their collected firewood not being there by the time they return to collect it. An NDO INDI explains how she occasionally pays a DO directly for help, and how she helps support her family without a donkey.

Case Study 2: direct hire of donkey and cart services 

Kali (RID41F) is a 41-year-old female NDO from Gia, with four children, two boys and two girls, all still at school. She is the second wife of her husband and refers to his first wife as her “rival”. The family own a mobile phone and a motorbike: they farm five acres and if they want to go anywhere, they walk. Kali controls how their overall income is spent: “I do whatever I can to feed my household, because my husband has two wives and so we the wives have to struggle on our own to support our husband to keep the home … anytime our husband fails to provide, my rival and I have to help. We, the women in the house [provide more income than our husband]”. Kali’s grandfather had a donkey when she was a girl that saved her a lot of time: she currently can “only boast of owning a fowl”. Kali is aware that owning a donkey would save the transport costs she occasionally pays out to DOs. “I must wait for the owner to finish working before helping [me]”. This is a disadvantage because “what I would have done with the help of the donkey, I couldn’t do that work and it affects my productivity level […] and draws my income earning backwards […] I would have wished [to] own a donkey but can’t afford one. […] The amount I get from the trading can’t even sustain my household well, not to talk of saving to buy a donkey”. She advised that she and her children carry firewood, farm produce and manure on their heads, but when asked about transporting produce to market she said: “The produce from the farm is not even enough for family consumption, let alone taking it to market to sell!” When asked about building or reconstructing their house Kali answered: “Sometimes when we can afford, we hire people to carry sand, water and other materials, or a donkey or a tricycle to carry the building materials. But if we can’t afford we have to carry the building material on our heads”. 

##### The Role of Children in Direct Income Generation Utilising Donkeys

A number of DOs during the first field visit, including Jalia, mentioned the role children play in household income generating opportunities using the family donkey. This was explored further in the second field visit. To use donkey(s) for income generation, households not only need a donkey cart but also a free family member to accompany it. DOs do not hire their donkey and cart out unaccompanied to ensure the cart is loaded properly, the agreed terms of the hire are fulfilled and payment collected. The most efficient method is for a child of at least 10 years of age to undertake this role, leaving parents free to continue their EADLs without interruption. This requires complex management skills juggling competing gendered production, reproduction, community [81] and income generation needs. Since September 2018, all children of both genders receive free, universal schooling in Ghana although it is only compulsory until the age of 14. Education provision appears to be highly valued by adults and children alike and children will only be taken out of school to attend a family funeral or if ill. Therefore, if at all possible, DOs will arrange the direct hiring of their donkey and cart during the afternoon or at weekends. As one adult male FGD respondent advised (FC001DM): “The issue of children working on the farm before going to school in the morning is no longer happening. You the parent can use the donkey to plough without children having to stop school to work on the farm. If you see children working on the farm, then it is Saturday”. It is important to bear in mind the possible social-desirability bias in all responses, especially those of the adults. 

Adults will not forego hiring out their donkey and cart during school hours, even if it means they themselves have to accompany the equipage, but as Jalia’s comments above indicate, optimising income generation from the family donkey via the direct hire method can depend on having children of the “correct” age. For maximum efficiency across the household’s working day however, families in both communities utilise children in the accompanying role. As noted, adults deem children to be mature enough at 10 years of age to be responsible for a donkey (Figure 4), although it appears the donkeys may sometimes have other ideas. One little boy in Gia during the dry season said he didn’t find that his donkey reduced his workload: “My donkey doesn’t help me, because when I try and use it to work, it refuses” (RID45). Interestingly, children use their donkeys in gender-specific ways for domestic EADLs, but working with their donkey when it is hired to a third party is gender neutral (Figure 4, column five). 

#### 3.2.3. Speculative Sales as a Means of Generating Income from a Donkey and Cart (Indirect)

The first indirect method of how donkeys help generate income, using firewood as an example, is to collect timber from the bush, bring it home and then to sell speculatively to anyone who needs wood (Method 3, Table 2). This has been entitled speculative sales. DOs speculatively sell to any person who can afford to pay the fee they charge. Female NDO respondents who cannot afford to purchase firewood garnered by someone else, either for domestic use or for “commercial” petty trading activities like pito brewing, will most likely collect their own wood. With no spare cash to afford this service they will carry it back to their household on their head, possibly with the help of their children, especially their daughters. A female DO INDI from Gia not only illustrates this method in more detail in relation to markets, but also indicates how DOs can be directly approached by an NDO like Kali to hire various services of their donkey and cart:

Case Study 3: Selling speculatively 

Maiara (RID28F) is a 36-year-old with two children: one boy and one girl who are both at school. Educated to secondary level she and her husband farm 1.5 acres. When asked she said that it is her husband who owns the donkeys as he bought them for her, but later, with regard to assets she said “we help each other because is for all of us and we need to help each other to make life smooth for us. So we both own the assets in the house”. The most important economic activity Maiara undertakes to earn income for her household is “the production of malt for pito breweries. “It’s the donkey that carries the water for me to prepare the malt. It also transports sorghum from Paga market to the house […] not having one would make life difficult for me and my household, so it is compulsory for me to own a donkey […] “It helps in the preparation of the malt and I make money from the sale of the malt and people do hire the services of my donkey to carry their stuff, but is not all the time […]. It also carries vegetable leaves from the bush to market for sale […]. If [I] am supposed to use two hours to perform an activity in a day, with the help of the donkey I can use at least one hour [and] I use that time to do my house chores”. She advised that: “the donkeys help me to save the cost I would have incurred to transport my goods from the farm to the market and all lot of things. But for hiring the donkeys out to make money is just a little amount of money I get because it’s my friends and relatives who begs the services of my donkeys and wouldn’t pay anything to me. What I get mostly from them as appreciation is thank you, may God bless you”. Maiara added that she and her husband do not have a routine as to “how we both use the donkeys. We will consider the most pressing activity first, irrespective of whether mine or his. We accept both views and we understand ourselves. We don’t quarrel over the use of the donkeys”. 

#### 3.2.4. Speculative Collection as a Means of Generating Income from a Donkey and Cart (Indirect)

A second way that DOs can make money indirectly using their donkey and cart is to collect firewood to sell “commercially” to specific petty traders, entitled speculative collection (Method 4, Table 2). Rather than collecting wood to be sold to any person who needs it, this is where a particular target audience is kept in mind by the DO when collecting wood to sell to people who need it to successfully carry out their own petty trading, or “commercial” opportunities. These include those, primarily women, who brew pito for sale or who cook at the market on open stoves to provide sustenance to the traders and buyers throughout the day. These purchasers also have the option of hiring a donkey and cart directly to collect firewood as outlined in Section 3.2.2 and Section 3.2.3, to ensure they have enough supplies for their requirements. A male DO INDI from Gia illustrates this method in more detail:

Case Study 3a: Speculative collection 

Kahiau (RID27M) is a 67-year-old male DO of Traditional African Belief (TAB) who completed primary education. One adult son “is around” and manages the donkey and the household owns a mobile phone, a radio, a bicycle and a motorbike. He, his wife and son farm 5 acres, producing peppers and other staple crops. His family-owned donkeys when he was a child, although in “those days, they didn’t use donkeys for any activity: it was just kept at home […] by our fathers to serve as bride price and to use for sacrificing to the gods”. Donkeys help him “make money to support my family, using them for ploughing, conveying manure and firewood to sell […and] any time I don’t have money I use the donkey to work and earn money […]. So the donkey helps a lot. [In addition] I also supply firewood to pito breweries and also supply wood for people to use for building of mud houses. […] When I am to go for firewood in the bush I have to wake up very early in the morning, like 5 a.m., because the bush is far […] I sometimes get home in the late afternoon, So I can say that the donkey saves me time at least 3 to 4 h. But the donkey “does a lot of activities […] contributing […] 40% for chores and 60% for income”. Kahiau said that it was his wife who controlled how their overall income was spent “because she knows what to buy for the upkeep of the house. But we both put our resources together to support our household”. When asked what they would do if they both needed their donkey at the same time he added: “We will come into agreement and allow one to use it because all is going to benefit the household [laughter]!” 

Case Study 3b: Indirect hire of donkey and cart, purchasing speculatively 

As an NDO brewer of pito, Selma (RID38F) is a potential customer of DOs utilising indirect methods 2, 3 and 4, although it seems she tries to harvest the firewood herself and hires a donkey and cart to transport the fuel home, rather than pay for two journeys. She is 63 years old, from Fielmon, with one son still at school. Her family own a mobile phone, a radio and a bicycle. Although they own seven acres of land, which is three miles from their home, they can only farm four: “we don’t have enough funds to farm all and we are also old: we don’t have the energy to farm [it] all […]. I brew pito, rear livestock and sell soybeans”. Selma can’t afford to purchase a donkey, although she believes that “the work of donkeys assists women to be able to do their work with ease. Now, there are not [many] differences between men and women in terms of work. The donkey does much of the work nowadays”. She also thinks that a donkey could help her a lot in her economic activities: “As at now, if I brew the pito and no one is there to assist me to carry, it would be difficult carrying them to the market. If I have a donkey, I would use it to carry the pito to the market … [instead of on my head] ... It would help me in earning income”. When asked, Selma said that her “husband would have made the most money” if they owned a donkey. This is because whoever used the donkey to work would have to give the money earned to my husband since he is the head of the household. Women don’t own a house; men control the household income. The hire of donkeys occasionally helps Selma: “No, I carry everything. If I would engage a donkey, I have to pay for the services. [ … I engage them] when we start harvesting firewood. If I harvest enough firewood in the bush, I hire the services of the donkeys to cart them home for household use. In terms of getting firewood for the pito brewing, I purchase them from those sending them to the market to sell”. 

As noted above, a number of options are open to both DOs and NDOs when the services of donkeys and carts are hired. It is interesting to consider Selma’s last point regarding her purchase of firewood to execute her trade in pito from “those sending them to the market to sell,” in conjunction with Kahiau’s comment “I also supply firewood to pito breweries”. These comments indicate that some DOs may build up a regular clientele of NDOs—or NDOs regularly utilise the services of the same Dos—who need transport services for their non-domestic, or “commercial” petty trading. Further research into the speculative collection methods of income generation via a donkey and cart is therefore recommended.

#### 3.2.5. Generating Income through the Sale of Donkey Intestine Soup (Indirect)

A further indirect method where either DO and NDO women can make money from donkeys was unique to the study site, Gia. This has been called donkey intestine soup (DIS, Method 5, Table 2). Donkey meat is increasingly being eaten in this community, with DIS becoming more popular, or “fashionable,” as a key informant described it. None of the female respondents in Gia earned money this way, so no further details, including costs were obtained. However, women who had arrived on bicycles waiting to purchase donkey intestines were observed by the research team at one slaughterhouse in Doba [50,51] and some were seen hawking the actual soup nightly on the return journey from Gia to Navrongo. This method is location/culture-specific and relies on donkey meat being available from butchers rather than owning one, hence why it is open to both NDOs and DOs.

Case Study 4: DIS 

Shapur (RID21M) is a 38-year-old male of Traditional African Belief from Gia with five children: three girls and two boys who are all in school, except the youngest girl. He completed the survey and a contextual interview, revealing that he had spent some time living in the city of Accra. His family owns a mobile phone, a radio, a TV, a bicycle and a motorbike and farm 4.5 acres of the five they own. He leaves his 0.5 acre for dry season gardening (“the cultivation of vegetables like pepper, tomatoes and garden egg”). Shapur reported in both the wet and dry season that he worried all the time about both not having enough income for the family’s needs and not having enough to eat, with food being scarce in March, April, May and June. He believed their donkeys contribute to any disposable income they make by reducing expenditure on transporting manure and goods in and out of the market: when asked to put a value on this input, Shapur described it as “priceless”. With knowledge from donkey butcher colleagues in Navrongo, he advised that: “100% of the [donkey] meat is sold in Kumasi and 100% of the intestine is sold locally to women here. The women use the intestine to prepare light soup and sell to earn money. It’s a form of business to them”.

#### 3.2.6. Donkey Ownership Generates Income Benefits through the Saving of Time (Cashless Indirect)

These case studies reveal that not only do DOs utilise a range of different methods open to them to make money but that saving transport costs is an additional benefit to owning donkeys that DOs appreciate. Although it means that this is a cost that DOs do not have to spend in comparison with NDOs, it is obviously not a method of making money per se. The time donkeys save DOs on a range of activities also frees them up to earn income they otherwise could not, including the ability to take on paid work. This quote by Esosa, a female DO INDI from Gia illustrates that even if they never hire their donkeys out, owning one “saves me time to engage in a day job like sowing on someone’s farm for money” (RID29F), labour that Kali would pay GH¢15 (£1.92) a day for. A corollary is that DOs, saving themselves time carrying produce to and from market can carry more in one donkey cart than one female NDO can on her head, probably even with help from her children. This enables DOs to make more money in a shorter time period. For these reasons saving time has been included as an indirect method of generating income, illustrated by a DO INDI from Fielmon: 

Case Study 5: Freeing time 

Abi (RID24F), a 30-year-old from Fielmon has six children at school and farms 21 acres with her husband. They decide together how to spend their overall income. However, although her husband owns the donkeys Abi controls the money earned from them “because I am working with the donkeys and I decide what we should use the money to do [ … ] I only sell [ produce ] when I am in need of money […] My main economic activity is farming but if you farm you don’t have money to purchase your basic items, you have to engage in petty trading to take care of that,” which in Abi’s case involves using her donkey to collect firewood to sell and selling charcoal. They purchased one female donkey, which has since reproduced, because “without the donkey, possibly we wouldn’t have always had food to eat […] our yield would have been very small to take care of our household food security. The donkeys are used to increase the farm yield […] helping me to feed my family […] Ploughing 21 acres of land is not easy. If it was a tractor or labour, they would have charged us GH¢100 (£12.80) [per acre…] We wouldn’t have been able to pay […The donkeys] also help because I use them to cart goods to the market, the income from the marketing activities is used to take care of the household’s basic needs”. When asked if she would sell her donkey, Abi replied: “I would never sell my donkeys if they are not very old or sick. Ei!! Sell my donkey and do what? What would I do to feed my family? I don’t have a female child to support me. The donkeys are helping me”. 

### 3.3. Income Generated from the Use of Donkeys

Table 3 shows that the amount of money each DO can earn via their donkey varies. Maiara estimated that “people do hire the services of my donkey to carry their stuff, but is not all the time”. She potentially earns an additional ~520–1040 GH¢ (~£72.80–145.60) per annum through hiring out her donkey, although as she points out this “is just a little amount because it’s my friends and relatives who beg the services of my donkeys and wouldn’t pay anything to me” [2,10,22,23]. This compares to Kahiau’s £6.48 for each load his donkeys carry for selling “speculatively”. He sells ~1 load on spec’ per week, generating an additional 2600 GH¢ (~£361) annually he can put towards the purchase of goats, for example. It is outside the scope of this paper to know whether the difference in monies earned per load is a result of potential differences in gender negotiating skills, and/or physical loading capability or whether a DO can actually make more money selling speculatively than hiring their donkey directly. However, these are interesting questions for future research.

Respondents were asked whether they could put a percentage on the time they used their donkey for chores versus their saved time undertaking marketing/income generating activities. In FGDs, women jointly concluded a 60% chores/40% income production split between these activities. The INDIs who undertook the Seven Daily Time Budget exercise were asked a similar question in order to triangulate results from the different research activities. In comparison, the four female INDIs estimated that their donkeys contributed between 30–60% of their household income: the uses they make of their donkeys to make money map to all of the benefits identified above as are available to them (Table 3). Even without using her donkeys to generate income directly, Jalia still felt that they contributed 30% of her total income. Esosa was the only person to publicly state that the key secondary role of her donkey is to make money: she uses every method available to her to do so, and recorded the largest contribution of donkeys to household income at 60%. Obviously, all DOs have the domestic and freeing time methods open to them as income benefits, even if they are not in a position to hire their donkeys out. Using the other four methods will be situated in time, place and circumstance. 

## 4. Discussion

The primary purpose of this research was to increase understanding of the contribution that donkeys make to the livelihoods of people in poor, rural communities, focusing on their use by women and children. Consistent with the literature, this study confirms the two key ways people use their donkeys in northern Ghana are mainly for ploughing and transport [35] p. 2. Nearly half (48%) use them to generate income by hiring them out [36] p. 1. The direct hire and cashless indirect methods have previously been documented, possibly because they are the most conspicuous [20,21,36,45,79]. However, this paper presents new evidence that firstly, the income generation methods using donkeys employed by DOs are more complex and multi-layered than has previously been described, with female donkey owners reporting that donkeys can contribute between 30–60% of their income. Not all methods are open to all DOs: they depend on family structure and local cultural traditions. Although based on a relatively small sample, these results broadly indicate that the greater the use of as many available methods of generating income from their donkey are available to a family, the greater the donkeys’ contribution to household income. A further study is therefore suggested to confirm and validate these findings. Secondly, children can play a key role in maximising household efficiency in generating income from a direct hire of the family donkey. This may be because it is the first time their donkeys have been discussed with children, or that the provision of free education for all children available in Ghana is facilitating their involvement in some way. Further research is needed to understand this modus operandi in more detail and identify what the children do with the time using their donkey saves them. 

DOs who don’t hire their donkeys out state that their donkeys contribute to household income through ownership alone. We have termed this the domestic method of how donkeys contribute to livelihoods, which is not well represented in the literature. However, the three indirect ways in which donkeys contribute to income benefits has yet to be understood. Speculative collection and selling contribute new evidence for the socio-economic benefits of donkey ownership. DIS is different in that the cash benefit to those who use it to make money resides in the value of a dead animal. This is why it is an income generating option for both DO and NDO women in UER. Although outside the scope of this paper, it should be noted that the increase in consumer demand for donkey meat in UER, from which DIS originates, plays a part in the growing pressure placed upon global donkey populations from the rising demand for the TCM, e’jiao [16,22].

NDOs have several disadvantages without having recourse to draught donkey power (DDP) and the range of income benefits that ownership of a donkey confers. Firstly, without a donkey to save them time, NDO’s equivalent EADLs will take longer. This will effectively reduce the time they have to hire themselves out to earn income, although those NDOs who own oxen can hire them out to plough in season. Farming activities, including ploughing manually, will take far more time than using donkeys and is very physically demanding. Transporting produce to the market to sell, either via headloading or a bicycle will inevitably take longer and/or decrease the amount of produce that can be carried as well. This reduces the amount of money that can be earned at the market in one session, in comparison to donkey owners. NDOs will then have to carry their purchases and unsold produce home again on their heads if they don’t have a bicycle, taking more time than DOs, unless they hire the services of a donkey-cart or motorised tricycle (MT) taxi to do so. If the sale or purchase of market produce is time critical, then NDOs who have to headload will need to get up earlier in the day to ensure a timely arrival at the market to secure a good pitch. Potentially, this also results in NDOs getting home later than DOs, resulting in an even longer day: again, especially for women because of the cultural and social norms surrounding the gender orientation of women’s EADLS, which includes marketing and petty trading. This precludes opportunities for finalising other EADLS in comparison to DOs. Selma and Kali’s case studies indicate that all these situations can be overcome through the hiring of a donkey and cart, plough, MT or oxen, but with the disadvantage of eating into any cash reserves they may have: cash that could be spent on alternatives. 

Secondly, and related to the first point above, the only recourse for NDOs to transport products without incurring costs is for them to be carried via the head, a bicycle or a hand cart. Kali and Selma’s case studies illustrate their dilemmas. As noted, this inevitably takes more time and results in a lot less product able to be carried than with a donkey and cart. More trips to collect firewood will then be required to keep fuel stocks at a usable level for domestic purposes, which takes longer if the only option is headloading. Donkey owners can stock up their wood supplies so that they may only have to go to the forest every few days: as well as reducing drudgery, this frees up considerably more time to undertake other EADLs and income generating activities that non-donkey owners just do not have, as seen from Kali’s case study (Section 3.2.2).

Thirdly, there are health disadvantages to non-donkey owners that arise from having to carry loads on their heads. If they have no spare cash or alternative transport available, NDOs have no option but to suffer axial stress through headloading, with its attendant disadvantages [73,74,75,76,77,78]. Both female and girl DO and NDO respondents spontaneously mentioned pains that can arise in their neck, head and chest through having to headload. DOs recalled these pains prior to owning a donkey, with one FGD respondent stated she had nearly become a “hunchback” prior to purchasing hers (RID50F2018). Male FGD respondents implied that women have actually lost the ability to carry loads on their head through lack of use over time: “As we have used the donkeys for such a long time, the women are now lazy to carry goods or water on their heads” (RID52M2018), although one man appeared more sympathetic: “Some of the women have to go to the stream [to collect water] while carrying babies, they come back complaining of chest pains because of the carrying of the water, but with the donkey, they do not have to carry this water on their heads so is in a way ensures we are healthy” (RID53M2018). Domestic load carrying is regarded culturally as a low-status, female activity, the burden of which falls “disproportionately on women and children” [73] p. 90. Duncan (1997) estimated that in Ghana “women carry up to two head loads of 30 kg over an average of 5 km a day” [74] p. xv. In Fielmon and Gia this burden is more likely to fall on female NDOs of all ages, than DOs. 

Fourthly, the collection of wood and water for domestic purposes are the two EADLs that households need to undertake most frequently. As gender-orientated chores, these impact on the daily lives of women to a far greater extent than men. Without a donkey they impact most acutely on female NDOs. As forests closest to compounds and farms are cleared first, the distance required to access wood for domestic and “commercial” purposes increases [82,83,84]. Obviously the further away usable stocks are, the more time it will take to travel to and from them, with or without a donkey. This is an on-going process. DOs will still take less time to travel increasing distances than NDOs, and therefore will still save more time. This could partly help explain the development of the market for hiring out donkeys and carts seen in both communities. Interestingly, the majority (6/8) of NDO survey respondents would prefer to own a donkey than an MT because they perceive donkeys as much more useful than MTs. This was not just because of disadvantages in accessing forests, but also for perceived additional difficulties and costs in maintaining MTs on an on-going basis, in comparison with donkeys.

Around the world, women spend two to ten times more time on unpaid care work than men [85], which is a global issue and not unique to the DO and NDOs in Fielmon and Gia, nor to this region of Ghana [86,87]. Maiara (DO RIDF36), Abi (DO RIDF30) and Kali (NDO RIDF41), all mentioned having to earn money for their household, illuminated by Kali’s comment: “Anytime our husband fails to provide, my rival and I have to help. We, the women in the house [provide more income than our husband]”. As outlined by our female respondents, and supporting the literature, the majority of domestic and reproductive EADLs, such as combining fetching firewood, water and marketing with childcare still fall disproportionately on them, all of which “activities are generally exclusive to women, acting as severe time burdens on their productivity” [73] p. 108. They have to combine these activities with their monetised work, so it can be argued that generating income needs to be added as a fourth load to Moser’s (1989) triple burden of unpaid production, reproduction and community management concept [81]. 

Moser’s triple burden research [81], plus additional studies into gender time use and divisions of labour in agriculture, confirm the fact that rural women in poor households work longer hours than men, often shaped by traditional gender roles acceded to over time by “society” [87,88,89]. However, as outlined in their case studies, donkeys do not appear to reduce the number of hours per day female DOs work. They undertake a range of their other EADL activities, or paid labour during the time they have saved via DDP. NDOs have alternative means of generating income without a donkey, as evinced by Kali and Selma through the hawking of fish and the selling of petrol or pito, (or in the case of two male respondents through renting out their oxen to plough or working as a tailor). However, there is clearly a large productivity gap between female DOs and NDOs. Many of the EADLs undertaken by NDOs will take longer and involve more hard physical work without a donkey and, as noted by Kali, does reduce her productivity. This limits the income she can generate, with potentially negative impacts on her food security as well. Although donkey ownership may not reduce the quadruple burden of long days for female DOs, donkeys not only increase female DOs productivity, they also reduce their drudgery. This is illustrated from Selma and Kali’s testaments to their productive, reproductive community and commercial activities. 

As noted, this exploratory study is limited by a relatively small sample size and it is important to bear in mind the possible social-desirability bias in these self-reported responses. The study was constructed to try and minimise the effects of response bias, developing different research activities to triangulate the data. Despite these limitations we believe this research provides new insights into the contributions donkeys make to livelihoods in northern Ghana. Our results reflect those of Tuaruka and Agbolosu (2019) who also found that the key role for donkeys in the Northern Region of Ghana was transporting various goods. “The least role was for income generation” [44] p. 1. They are also consistent with Braimah et al., (2013) who concluded that the reduction in drudgery and saved time, especially for women, were key to the value ownership of a donkey provides. Our results provide important insights into these conclusions. Only one of the four burdens women have to contend with on a daily basis is to make money to support their families. (Kali: Section 3.2.2). Donkeys reduce their drudgery across all four burdens nearly every day, including women’s reproduction, production and community responsibilities. We argue this is the reason why reduced drudgery and saved time are more important to DOs than income generation.

However, the study demonstrates that although hiring out donkeys to make money is not key to why respondents own donkeys, nor to why NDOs would like to own one, the income generation methods using donkeys employed by DOs are more complex and multi-layered than has previously been described. Just owning a donkey provides a range of income benefits to owners. These include: (i) a range of options to make money, including the time donkeys save households, enabling other income-generating activities to be undertaken. This is illuminated by Esosa being able to accept a day’s sowing for another farmer; (ii) potential health gains from minimising axial stress from headloading; (iii) why three of the methods DOs have at their disposal to make money from their donkey have remained invisible and (iv) children can also play an important role key in the efficient deployment of income-generating opportunities through the direct hire of donkeys. 

Geiger et al., (2020) conclude that “assessing the socioeconomic value of donkeys within different locations within the same area or country is critical, rather than assuming that similar views are held between compatriots” [45] p. 12. It is unlikely that all our results are fully generalizable to other poor, rural donkey-owning communities. This is especially true of the importance of efficient resource allocation using children, as currently this is the only empirical investigation into the relationships between children and their donkeys. These preliminary findings signpost further research opportunities to analyse the value of donkeys and their economic contributions to poor, rural communities. These include studies aimed at better understanding the role donkey ownership plays in minimising axial loading and other aspects of health and wellbeing; whether donkeys and children contribute to similar income generation strategies in countries where free universal schooling to both genders is not provided; whether owning a donkey contributes to longer working days for women, especially productivity differentials between DO and NDO women, including improved food security; which institutions will provide credit in relation to male and/or female donkey ownership and investigations into potential gender differences in sporadic and daily workloads across the agricultural season. 

## 5. Conclusions

Donkey populations are under threat. However, donkeys are important to their owners and donkeys’ contributions to livelihoods are more complex than previously understood and documented in the literature. The ownership of donkeys confers up to six different income benefits in comparison to non-donkey owners, depending on location. Female donkey owners reported that donkeys can contribute between 30–60% of their income. Children of both sexes play a role in the efficient deployment of one of these income generating activities. 

## Figures and Tables

**Table 1:** Children’s Focus Group Participants.

Study Site			No. of Respondents	Girls	Boys	Age Range (years)	Mean Age (years)	Family Owns 1 Donkey	Family Owns 2 Donkey	Family Owns 3 Donkey
Fielmon	Mixed sex FGD	December 2018	12	3	9	10–16	11.58	7	4	1
	Girls only	June 2019	8	8		12–15	13	4	4	
	Boys only	June 2019	8		8	12–15	13.2	8		
Gia	Mixed sex FGD	December 2018	11	4	7	10–16	12.81	0	8	3
	Girls only	June 2019	7	7		10–13	12	2	4	1
	Boys only	June 2019	8		8	10–14	13	2	4	2

**Table 2:** The six income benefits conferred through donkey ownership.

Method of Income Generation		Description	Potential Earnings Per Hire (GH¢)	Illustrative Participant Quotation	RID
1.	Domestic (Cashless Indirect)	No income generating activities *per se.*	Not applicable, but saves costs.	Still contributes to household income, through not having to hire in transportation and ploughing assistance - information provided by Jalia (DO). Also saves time, itemised separately under 6.	25
2.	Direct Hire (Direct)	Hire directly for an EADL.	10.00–20.00 GH¢ (£1.40–2.80).	Approximate weekly income from hiring out her donkey, costs quoted by Maiara (DO).	28
3.	Speculative Selling (Indirect)	Collect a load of wood with no one in mind to sell it to, to sell 'on spec' to anyone who needs wood, for any purpose.	“Costs 5 GH¢ (£0.70) twice per week to hire a donkey to cart firewood back from the bush.”	Costs to hire a donkey and cart to collect firewood she has cut and collected in the bush, quoted by Selma (NDO). The only alternative is for her to carry it home on her head.	38
4.	Speculative Collection (Indirect)	Collect firewood to sell “commercially” to specific petty traders. Rather than collecting wood to be sold to any person who needs it, a particular target audience is kept in mind by the DO when collecting wood.	50.00 GH¢ (£6.95) per day.	Anytime I don’t have money, I use the donkey to work and earn money at the end of the day. I can make at least 50.00 GH¢ and I can use that money to buy a female goat and it will reproduce” Kahiau (DO).	27
5.	Donkey Intestine Soup (Indirect)	Selling donkey intestine soup.	Unknown.	An option available only in Gia. Sharpur (a DO) reported women collecting donkey intestines at the slaughterhouses, making soup from them and then selling the soup, as “a business opportunity.”	21
6.	Freeing Time (Cashless Indirect)	Saving time, which can be converted into money, through paid employment.	"We usually pay GH¢ 15 (£2.10) per person on [a] daily basis."	Costs provided by an NDO Kali (NDO 41) who reported: “We pay people to plough, sow, and even weed on our farm," to the benefit of Esosa, a DO (29) who advised that owning a donkey "saves me time to engage in a day job like sowing on someone’s farm for money.”	41 and 29

**Table 3:** Donkeys’ contribution to household income in northern Ghana.

Unique ID	Study Site	Gender	Respon-dent *	Key EADLs Using Their Donkey	Income Generating Methods Used	Donkey’s Contri-bution to HH Income	Time Their Donkey(s) Saves Her/Him
29	Gia	Female	Esosa	Income generation was the second key reason why she owns a donkey as it is the main source of her income.	Domestic; Direct Hire; Speculative Selling; Speculative Collection and Freeing Time.	60%	“I can go to the market to sell my vegetables after using the donkey to carry out carting work/job”.
28	Gia	Female	Maiara	Brews pito to sell and does hire out her donkeys for income.	Domestic; Direct Hire; Speculative Selling; Speculative Collection and Freeing Time.	40%	2 h a day
24	Fielmon	Female	Abi	Domestic use, saved time and hiring out for income through her children. Currently, only her boys are old enough to assist in hiring donkey out: her girls are too young.	Domestic; Direct Hire; Speculative Selling; Speculative Collection and Freeing Time.	40%	6 h a day
25	Fielmon	Female	Jalia	Domestic use and saved time only: currently, no children old enough to help with hiring their donkey out.	Domestic; Freeing Time.	30%	<1 h a day (6 h per week)
27	Gia	Male	Kahiau	Domestic, farming and gardening use, plus conscious breeding of females to minimise re-purchase costs.	Domestic; Direct Hire; Speculative Selling; Speculative Collection and Freeing Time.	~50%	“Much time is saved for my household to perform other works. The saved time I use it to work on my garden.”
26	Fielmon	Male	Kaif	Domestic and farming chores and occasionally rents out his donkey and cart to carry firewood for a fee.	Domestic; Direct Hire; Speculative Selling; Speculative Collection and Freeing Time.	40%	Saves men 5 h per week; women 5 h per week; children 10 h per week

Data from Seven Daily Time Budgets (7DTBs). * Pseudonyms.

## Data Availability

The datasets generated for this study will not be made publicly available. Anonymised data sets relating to EARS, QBA and all livelihood surveys and interviews can be made available on request to the corresponding author. Full interview transcripts cannot be made available as participants did not explicitly give consent for this and full privacy was promised. URLs/accession numbers/DOIs will be available after acceptance of the manuscript for publication.

## Supplementaly Materials

The following are available online at https://www.mdpi.com/article/10.3390/ani11113154/s1, Figure S1: Copies of all research instruments, Figure S2: Origi-nal_NVivo_visualisation_upon_which_Figure_4_is_based.
